# Open, Randomised, Controlled Study to Evaluate the Role of a Dietary Supplement Containing *Pelargonium sidoides* Extract, Honey, Propolis, and Zinc as Adjuvant Treatment in Children with Acute Tonsillopharyngitis

**DOI:** 10.3390/children12030345

**Published:** 2025-03-10

**Authors:** Fabio Cardinale, Dionisio Franco Barattini, Alessandro Centi, Greta Giuntini, Maria Morariu Bordea, Dorina Herteg, Luca Barattini, Cristian Radu Matei

**Affiliations:** 1 AOU “Policlinico-Giovanni XXIII”, Ospedale Pediatrico Giovanni XXIII—Università di Bari, 70126 Bari, Italy; 2 Opera CRO, a TIGERMED Group Company, 300209 Timisoara, Romania; barattini@operacro.com; 3 Pediatrica Srl, 57121 Livorno, Italy or alessandro.centi@pediatrica.it (A.C.); rd@pediatricaspecialist.it (G.G.); 4 CMMF Dr. Morariu Bordea, 300425 Timisoara, Romania; cabinetmbmorariu@yahoo.com; 5 CM Dr. Herteg, 300254 Timisoara, Romania; herteg.dorina@yahoo.com; 6 TIGERMED Italy, Corso Buenos Aires, 6, 16129 Genova, Italy; personal@lucabarattini.com; 7 CM Dr. Matei, Otelu Rosu, 325700 Caras-Severin, Romania; dr_matei_cristian@yahoo.com

**Keywords:** dietary supplements, tonsillitis, *Pelargonium sidoides*, honey, propolis, zinc

## Abstract

Background/Objectives: A common reason for a pediatrician’s visit is acute tonsillopharyngitis (ATR), which is usually caused by viruses. A dietary supplement comprising *Pelargonium sidoides* extract, honey, propolis, and zinc was proposed as an effective adjuvant for the management of respiratory tract infections. The study aimed to determine the efficacy of this dietary supplement in conjunction with standard of care (SoC) compared to SoC alone, in a pediatric population affected by ATR. Methods: This open randomized study (registered on ClinicalTrials.gov: NCT 04899401) involved three Romanian sites specialized in pediatric care. The primary endpoints were changes in Tonsillitis Severity Score and the number of patients failing to respond (evaluating the use of ibuprofen or high-dose paracetamol as a rescue medication). One hundred and thirty children, distributed into two groups, were enrolled and treated for six days. Results: The results showed an overall better performance in terms of efficacy of dietary supplement + SoC, compared to SoC alone, with lower total Tonsillitis Severity Score ratings on day 6 (*p* = 0.002) and lower sub-scores related to erythema and throat pain on day 6. No adverse events were reported. Investigators found compliance to be optimal. Conclusions: The administration of the dietary supplement + SoC in pediatric patients with ATR was found to be safe and superior to the administration of SoC alone in terms of efficacy. The results confirmed that the tested dietary supplement is an optimum effective adjuvant in the treatment of respiratory tract infections and is suitable for the daily clinical practice of pediatricians.

## 1. Introduction

### 1.1. Acute Respiratory Illness and Antibiotics Prescription

Acute tonsillo-rhinopharyngitis (ATR) is an acute infection of the nasopharynx/nasal cavities, palatine tonsils, or both. They mainly affect children and adolescents [1] and are one of the most common reasons for consulting a pediatrician. Diagnosis is generally made clinically, with symptoms including sore throat, cervical lymphadenopathy, dysphagia, and fever. In a few cases, the diagnosis should be supported by culture or rapid antigen tests. According to common opinion, the vast majority of ATR cases are caused by viral infections, including adenoviruses, Epstein–Barr virus, human boca virus, influenza, para-influenza, rhinoviruses, and enteroviruses including Coxsackie viruses [2]. Bacterial etiology, therefore, represents a much smaller percentage, estimated by various authors to account for no more than 30% of all cases of ATR [2]. Obviously, only ATR of bacterial origin is an indication for antibiotic therapy. As indicated in guidelines [2,3,4], the use of antibiotic medications is not recommended for pediatric patients suffering from suspected viral respiratory diseases (bronchitis, pharyngitis, sinusitis). Despite the overall decline in antibiotic prescriptions for children in many countries, this unnecessary administration remains high. The risk of individual antibiotic resistance, potential adverse events (AEs), and an increase in social care costs represent a real and actual danger. In Italy, the number of children treated with antibiotics at home is four times that in the United Kingdom (52% vs. 14%) [5]. Acute bronchitis, non-specific upper respiratory infection, common cold, and acute cough are conditions in which antibiotic therapy is not indicated for the predominant viral etiologies [6]. In more than two million pediatric office visits each year, acute bronchitis is diagnosed, and doctors prescribe antibiotics for approximately 70% of the cases. As observed [6] using the diagnostic clinical criteria for acute otitis media (AOM), sinusitis, and pharyngitis, clinicians should be able to evaluate the etiology correctly and prescribe the appropriate treatment. Only in a few cases is antitussive therapy (e.g., dextromethorphan) suggested [7], although it must be remembered that it is not advisable for children under the age of 6 years. Consequently, research is focused on new products that can be used in daily clinical practice by pediatricians to treat viral-induced respiratory diseases, reducing the improper use of antibiotic therapies.

### 1.2. Dietary Supplements as Adjuvant Treatment in Upper Respiratory Tract Infections

According to the available scientific literature, *Pelargonium sidoides* extract has been shown to be an effective beneficial option for respiratory diseases [8,9]. In particular, its appropriateness and effectiveness have been demonstrated in the treatment of pediatric populations, both in acute non-streptococcal tonsillopharyngitis compared to placebo [10], and for the treatment of acute bronchitis beyond the strict indication for antibiotics [9]. Additionally, other substances, including honey, propolis, and micronutrients such as zinc, are currently used as dietary supplements (DSs) in clinical practice for pulmonary diseases and are supported by a substantial body of the literature [11,12,13]. In particular, a Cochrane meta-analysis on honey published in 2018 included six clinical trials involving 900 children and demonstrated that honey effectively alleviated cough symptoms in comparison with no treatment or diphenhydramine, but showed no improvement over dextromethorphan [11]. The anti-inflammatory effect of honey has been correlated with the decrease in free radicals found at the infection site. Honey also showed an improved immune system and also enhanced neutrophils, B cells, and T lymphocytes [14,15]. A systematic review showed that propolis is a safe DS with a favorable effect on glutathione peroxidase, glutathione, and total antioxidant capacity levels. When oxidative stress is a fundamental cause, these findings allow propolis to play a role as an adjuvant therapy [16]. Studies have demonstrated a significant improvement (i.e., a decrease in days to remission) with the use of propolis in uncomplicated bacterial and viral upper respiratory tract infections (URTIs) [17]. Recently, patients affected by chronic obstructive pulmonary disease (COPD) were treated with a composite of propolis and N-acetylcysteine in randomized double-blind trials [18]. These studies demonstrated an improvement in symptoms and quality of life (QoL) and a decrease in the frequency of COPD exacerbations in treated patients. During the recent pandemic, propolis has also been tested in randomized placebo/standard-care controlled trials [19]. This dietary supplementation showed limited, but statistically significant, beneficial effects in improving the signs and symptoms of COVID-19 (shortness of breath, dry cough, sore throat, and chest pain). A comprehensive systematic review [20] evaluated data from 8.526 treated patients and demonstrated the beneficial effects of zinc supplementation in reducing the average duration of the common cold. In the same review, the majority of the 15 studies (out of 34) focused on prevention failed to demonstrate a positive effect of zinc on the prevention of colds, and an increase in the number of non-serious adverse events (SAEs) was noted in the patients who received the supplement. The positive results in this important review of zinc supplementation and the fact that it was often used in conjunction with other treatments underscore that zinc can be considered a useful therapeutic tool in clinical practice and that physicians can safely administer it in conjunction with various therapeutic products.

### 1.3. Aim of the Study

These considerations have led Pediatrica Srl (Livorno, Italy) to develop the dietary supplement PediaFlù^®^ (DSPP) oral solution, as an adjuvant tailored specifically to the pediatric age for the well-being of the respiratory tract. DSPP is composed of the following: *Pelargonium sidoides* extract (Pelagon P 70™), honey, a formulation of propolis (PropolNext^®^ Plus), and zinc. The DSPP product is currently available in Italy and other European countries as an adjuvant for seasonal diseases. In the present clinical trial, we have tested the DSPP product to confirm its effectiveness, which has been proven in the clinical practice of hundreds of pediatricians. Therefore, we planned this study to assess the efficacy of a six-day course of DSPP in conjunction with standard of care (SoC) treatment, in ameliorating ATR symptoms in pediatric patients affected by ATR, in contrast to patients receiving SoC exclusively. We have recently performed a preliminary and partial evaluation of the trial results focused on the honey component by analyzing a limited subset of the parameters monitored during the study [21]. In contrast, the present article offers a comprehensive overview of the study outcomes and results with the aim of demonstrating the potential contribution of this trial to the enhancement of DSPP safety and performance. In addition, it provides pediatricians with the opportunity to autonomously assess whether the administration of DSPP in conjunction with SoC yields clinical benefits in children with ATR.

## 2. Materials and Methods

### 2.1. Study Design

The aim of the multicenter trial was to evaluate the efficacy and safety of adjuvant treatment of a commercially available DS containing *Pelargonium sidoides* extract, honey, propolis and zinc administered with SoC versus SoC alone to improve the symptom severity of ATR in a population of pediatric patients. This study used a randomized, controlled, parallel-group superiority design. We followed the methods described by Fabio Cardinale et al. [22] available at https://www.protocols.io/view/a-randomized-open-controlled-study-to-evaluate-the-cmtqu6mw.html (accessed on 6 December 2023). The primary objectives of the trial were to evaluate efficacy through changes in the Tonsillitis Severity Score (TSS) and assessment of rescue medication administration, which was considered a treatment failure, and safety, as measured by means of the incidence of adverse events (AEs) and serious adverse events (SAEs) during the duration of the study. The secondary objectives included overall symptom improvement, overall safety during the study, and compliance.

The three participating sites were Romanian private clinics specializing in pediatric care located in the Timisoara region (#01 Cabinet Medical Medicina de Familie Dr Morariu Bordea, #02 Cabinet Medical Dr Herteg Dorina, and #03 Cabinet Medical Dr Cristian-Radu Matei). The local Independent Ethics Committees approved the trial on 27 April 2021 (sites #01 and #02) and 23 April 2021 (site #03). The three sites were coordinated by the Department of Clinical Trials of the University of Medicine and Pharmacy Victor Babes in Timisoara, Romania. The sponsor charged a Contract Research Organization (Opera CRO, Timisoara, Romania) to perform the activities of monitoring, data management, and statistical analysis. On the other hand, the sponsor designated Prof. Fabio Cardinale (University of Bari, Italy) as the scientific coordinator of the study. In this role, he ensured the application of ethics requirements and the finalization of the protocol and shared the scientific information of the tested product with the investigators. The sponsor limited its activity to sending the quantity of DSPP to perform the trial to the sites and to offering a partial grant.

The trial was conducted in accordance with the principles of the Declaration of Helsinki and in compliance with the International Conference on Harmonization, Good Clinical Practice, and Italian and Romanian regulations. Prior to enrollment, all participants provided informed consent in writing, and no financial incentives were offered. This trial was registered in clinicaltrials.gov (NCT04899401) and the protocol was deposited in protocols.io [22]. The protocol description was recently published [23].

### 2.2. Participants

The patients were male and female children between the ages of 3 and 10 years old, suffering from ATR with a TSS score ≥ 8 for no more than 48 h. They also had negative results for the rapid tests for Group A beta-hemolytic *Streptococcus* (GABHS) and Severe Acute Respiratory Syndrome Coronavirus 2 (SARS-CoV-2) infection and negative nasal and/or pharyngeal exudate culture. Exclusion criteria included signs of follicular or lacunar angina, increased hemorrhagic diathesis or chronic diseases, having had more than two episodes of tonsillitis in the past 12 months, or close contact with SARS-CoV-2 infected persons within ten days from symptoms onset. Patients with known or suspected hypersensitivity to trial products, those who have been treated with antibiotics within four months prior to enrollment in the study, those presenting a mandatory indication for therapy with antibiotics, or those currently in treatment with agents potentially influencing the trial outcomes or having interactions with the trial products were also excluded. An additional exclusion criterion was participation in another clinical trial within the previous three months.

The investigators trained the children before the study began, in the presence of their parents, to respond correctly to the tests administered, particularly the Patient Global Assessment of Efficacy (PGAE). Parents were requested to maintain a record of their child’s temperature and document the administration of rescue medication in the patient diary throughout the study period. Furthermore, parents were instructed to return the unused medication at the final visit and to call the investigator in case of AE. The enrollment period started on 3 June 2021, and ended on 6 August 2021. The study was concluded on 12 August 2021.

### 2.3. Treatment and Allocation

Patients who met the eligibility criteria were enrolled and randomized into one of two groups in a 1:1 ratio: one group received DSPP in addition to SoC, whereas the other group received SoC alone. Randomization was conducted centrally by the CRO managing the study using a permuted block of random sizes. The package was GNU Library General Public License, v 2.1 from R statistical software v 3.5. Patients were allocated a unique 5-digit patient randomization number using an online web service (https://edc.operacro.com:4xx, which was available 24 h a day in a 1:1 group assignment ratio. Personnel involved in the allocation process were not involved in the evaluation of subjects in the study. It should be noted that no one (patient or parent) was blinded to treatment allocation. The tested DSPP was orally administered 5 mL × 3 times per day for six days for children under six years, and 10 mL × 3 times per day for six days for children over six years old. The DSPP contains Pelagon P-70™ (equal to 133.3 mg of *Pelargonium sidoides* extract per 100 mL), PropolNext^®^ Plus (equal to 7.7 mg of propolis extract per 100 mL), zinc (13.3 mg per 100 mL), honey (5.5 g per 100 mL).

SoC treatment included the following products:

- Benzydamine hydrochloride (throat spray 0.15%): According to the leaflet for children over six and below twelve: 4 sprays 2–6 times a day; for children under six years: 1 spray per 4 kg of body weight, a maximum of 4 sprays at once; the frequency was 2–6 times per day. Each spray was equal to 0.17 mL of solution.
- Paracetamol (120 mg/5 mL) per os, in case of >38.5 °C, 10 mg/kg/dose, per need every 6–8 h. Maximum dosage under 30 mg/kg dose.
- Nasal decongestion through hydration with fluids, aspiration of secretions, use of nasal irrigation with saline solution, nasal sprays with seawater or with an active ingredient (this last one was just a special indication from the doctor).

The use of products containing coumarin and antibiotics was prohibited during the trial. During the study period, the administration of paracetamol (>30 mg/kg) or ibuprofen (100 mg/5 mL) was classified as rescue medication. There were no restrictions on subjects’ previous treatments for conditions unrelated to this protocol.

Four visits were scheduled. During each visit, a thorough evaluation was conducted, encompassing an assessment of eligibility, a physical examination, an evaluation of disease, and a TSS score evaluation. Concomitant medications, AEs, and SAEs were also closely monitored and documented. At the screening visit (visit 1; day-2 to day-1), the presence of GABHS and SAR-COV-2 was ascertained, and the subjects’ medical histories and demographic information were documented. In addition, parents or legal guardians of subjects who met the inclusion criteria were requested to provide their signature on the informed consent form. At the baseline visit (visit 2, day 0), patient characteristics were recorded, and the tested products and diaries were provided. At the intermediate visit (visit 3, day 4 from recruitment), diary verification was performed. Final evaluations, symptoms, and compliance evaluations were performed at the end of the study (visit 4; day 6 from recruitment). The study products and diaries were also collected during this final visit.

### 2.4. Primary and Secondary Outcomes

The change in TSS [10] and the number of treatment failures between the two groups were used to measure the primary efficacy outcomes. The treatment failures were determined by analyzing the use of rescue medication.

The five subscores of the TSS are indicative of the characteristics of viral-induced inflammatory infections of the upper respiratory tract (non-GABHS), including sore throat, increased salivation, difficulty swallowing, and pharyngeal erythema. These items were scored at each visit by the investigator using a 4-point scale (3 = severe, 2 = moderate, 1 = mild, 0 = not present). Furthermore, the investigator assessed the patient’s fever as follows: <37.5 °C = 0; 37.5 °C to <38.5 °C = 1; 38.5 °C to <39.5 °C = 2; and ≥39.5 °C = 3. Consequently, the total TSS score for the five symptoms ranged from 0 to 15. A comparative analysis of the absolute change in score from baseline to final visit was performed between groups and within each group. TSS was assessed at each visit, and the total and sub-scores were evaluated.

The use of rescue medication (paracetamol >30 mg/kg or ibuprofen 100 mg/5 mL) by a patient was considered treatment failure.

The primary safety outcome was the incidence of AE/SAE during the trial.

The following secondary outcomes were recorded:

Investigator Global Assessment of Efficacy (IGAE): at the final visit, the investigators performed a global assessment of treatment efficacy using a 4-point scale (1 = excellent, 2 = good, 3 = fair, 4 = poor). The comparison was made between the DSPP plus SoC group and the SoC alone group.

PGAE: At the conclusion of the study, patients were asked to provide a global assessment of treatment efficacy using a 5-point scale designed specifically for children. The scale ranged from 1 to 5 as follows: 1 = very satisfied, 2 = satisfied, 3 = adequate, 4 = unsatisfied 5 = very unsatisfied. The comparison was between groups.

Investigator Global Assessment of Safety (IGAS): Investigators used a 4-point scale to rate the safety of the treatments. The scale went from 1 (very good safety) to 4 (poor safety). The evaluation was performed at the final visit.

Compliance: an evaluation of treatment adherence was conducted for the two groups to ascertain compliance with the administered treatments at the conclusion of the trial.

### 2.5. Sample Size and Statistical Analyses

The sample size was estimated in accordance with TSS, the primary outcome, and the findings of a previous trial with a similar design [10]. After a six-day treatment period, the minimal clinical difference between the two groups (DSPP + SoC versus SoC alone) was determined to be a two-point decrease in mean TSS. Therefore, in agreement with the established formula for calculating the sample size for a comparison of two means at a significance level of 5%, a power of 80%, and a minimally clinically important difference of 2 ± 3.85 points, it was established that 120 patients should be evaluated in the study. To obtain this number of patients, approximately 150 patients should be screened; this calculation included potential screening failure and estimated dropout patients.

All statistical analyses were performed using specific statistical software (version 4.1.0; R Foundation for Statistical Computing). The final analysis was completed subsequent to the conclusion of the trial, the resolution of all queries, and the locking of the database. The overall Type I error rate was maintained at 5%. All tests were two-sided. The statistical analyses were conducted on all patients who successfully completed the trial without a protocol deviation. An evaluation and comparison of the quality and completeness of the data collected set was also carried out. In the event that a patient had missing information for one or more variables, the missing data were not substituted. If a patient violated the inclusion/exclusion criteria, their data would be excluded from the analysis. The quantitative variables (i.e., demographic information) were described using the mean and standard deviation (SD) if they were found to be normally distributed. If these variables were found to be non-normally distributed, the median and interquartile ranges were used. Student’s *t*-test and Mann–Whitney U test were employed to perform a comparative analysis, taking into account the distribution of these variables. Additionally, factorial variance analysis was utilized to evaluate potential interactions between quantitative variables and linear progression models, with the objective of relating possible confounding bias to independent variables. Categorical variables were represented through frequencies and percentages, and a comparative analysis was performed using the chi-squared test.

## 3. Results

### 3.1. Patient Disposition and Characteristics

Of the 135 patients screened, 130 were considered eligible for the trial completion. Patients were randomly assigned to either the DSPP + SoC group (n = 66) or SoC alone group (n = 64). The 130 subjects enrolled in the study successfully completed the trial, thus being included in the intention-to-treat (ITT) and safety populations analysis. One hundred and twenty nine patients were included in the Per Protocol (PP) population; one patient belonging to the DSPP + SoC group dropped out.

**Figure 1:**
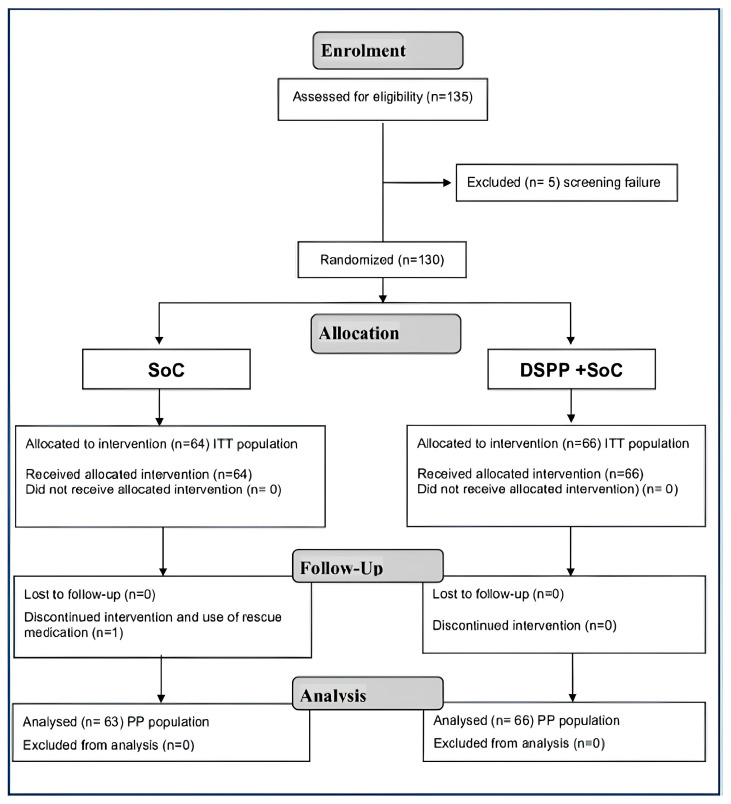
Consort 2010 flow diagram. Standard of care (SoC); dietary supplement PediaFlù^®^ (DSPP).

The reporting of this study conforms to the CONSORT Trials checklist: https://www.equator-network.org/reporting-guidelines/consort/ (accessed on 8 January 2024). Patient disposition is graphically displayed in Figure 1.

As shown in Table 1, there were no statistically significant differences (*p* > 0.05) in baseline demographic and anthropometric measures between the two groups.

### 3.2. Administered Treatments

**Figure 2:**
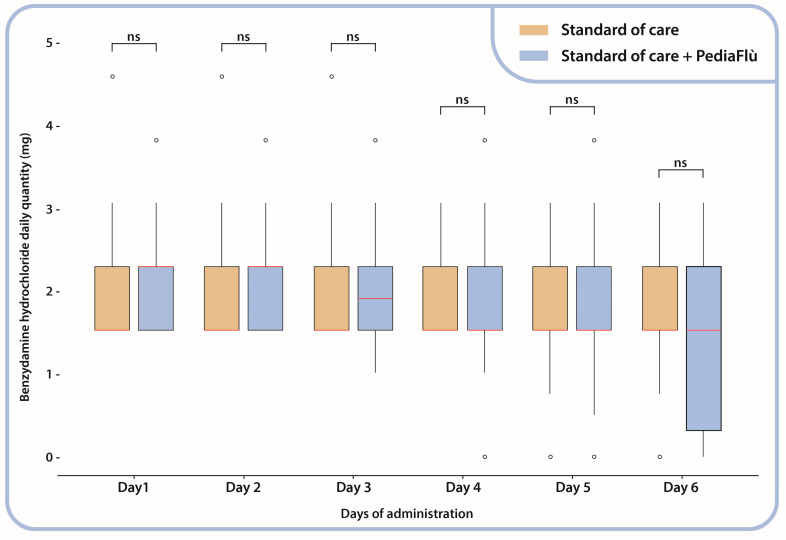
Benzydamine hydrochloride daily administration (mg); Mann–Whitney test between administration groups. Standard of care (SoC); dietary supplement PediaFlù^®^ (DSPP). ns: non-significant (*p* > 0.05).

All patients demonstrated adherence to the prescribed regimens for DSPP, low-dose paracetamol, and benzydamine hydrochloride. In particular, the analysis revealed no substantial intergroup differences with respect to the administration of benzydamine hydrochloride, as shown by the comparable number of doses and quantities (Figure 2).

In Table 2, a summary of low-dose paracetamol use as part of SoC during the 6-day study period is shown. A significant statistical difference in the mean number of doses administered was observed between the two groups (*p* < 0.01).

### 3.3. Efficacy Analysis

#### 3.3.1. TSS

**Figure 3:**
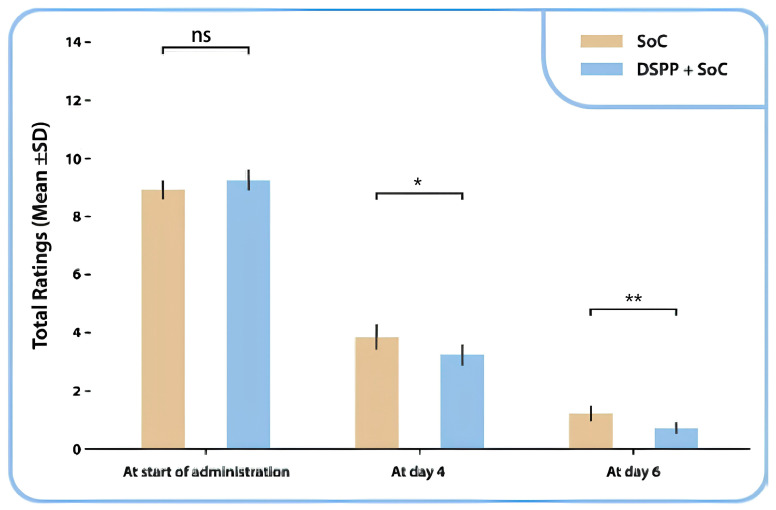
Tonsillitis Severity Score (TSS) total mean ± SD ratings between administration groups, at day 0, day 4 and day 6. Standard of care (SoC); dietary supplement PediaFlù^®^ (DSPP). ** *p* < 0.01; * *p* < 0.05; ns: non-significant (*p* > 0.05).

The variation in the TSS score from the initial visit to the final visit demonstrated that patients treated with DSPP + SoC exhibited reduced TSS ratings than patients treated with SoC alone (Figure 3). The Mann–Whitney test indicated a statistically significant difference between the two groups on days 4 (*p* = 0.034) and 6 (*p* = 0.002).

A statistically significant difference was identified between the two groups in the evaluation of TSS subscores for sore throat: mean (SD) 0.0 ± 0.2 in the DSPP + SoC group and 0.2 ± 0.5 in the SoC alone group, *p* < 0.001 and pharyngeal erythema: mean (SD) 0.6 ± 0.6 in the DSPP + SoC group and 0.9 ± 0.6 SD in the SoC alone group, *p* < 0.05.

#### 3.3.2. Use of Rescue Medication

The administration of ibuprofen was deemed necessary for a single patient (who belonged to the SoC alone group). This action was classified as a “rescue medication use” (1.6% of patients in the SoC alone group and 0.8% of the total patient population). The involved patient withdrew prematurely due to persistent symptoms and increased fever. No patients were administered a dose of paracetamol above 30 mg/kg/dose, which was considered in the protocol as the threshold for rescue medication.

#### 3.3.3. PGAE

**Figure 4:**
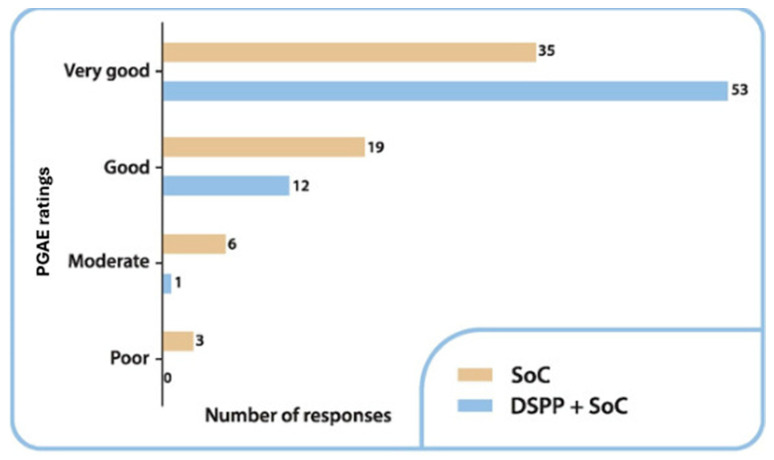
Patient Global Assessment of Efficacy (PGAE) ratings in the two groups at final visit. Standard of care (SoC); dietary supplement PediaFlù^®^ (DSPP).

The evaluation carried out using the PGAE on day 6 showed that the efficacy of the treatment was evaluated as very good by 80.3% (n = 53) of the patients in the DSPP + SoC group and 55.6% (n = 35) of the patients in the SoC alone group (Figure 4).

A statistically significant difference was identified (*p* = 0.013, as determined using the chi-squared test). On day 6, none of the patients in the DSPP + SoC group rated the treatment as poor, whereas 4.8% of the patients (n = 3) in the SoC alone group evaluated the effectiveness of the treatment as poor.

#### 3.3.4. IGAE

**Figure 5:**
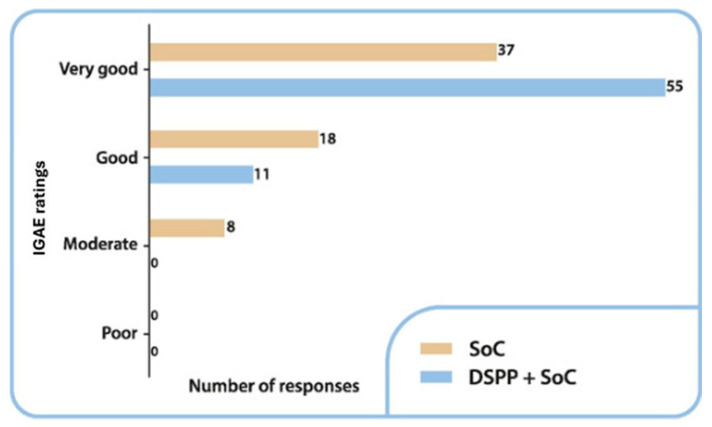
Investigator Global Assessment of Efficacy (IGAE) ratings in the two groups at final visit. Standard of care (SoC); dietary supplement PediaFlù^®^ (DSPP).

The IGAE among the groups is summarized in Figure 5. On day 6, treatment was evaluated as very good in 83.3% of patients in the DSPP + SoC group and 58.7% of patients in the SoC alone group. A statistically significant difference was identified between the groups (*p* = 0.04).

### 3.4. Safety Analysis

**Figure 6:**
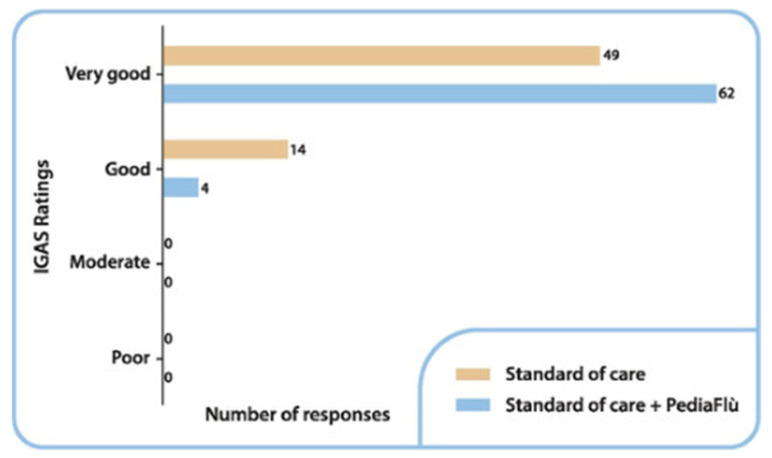
Investigator Global Assessment of Safety (IGAS) ratings by number of responses at final visit. Standard of care (SoC); dietary supplement PediaFlù^®^ (DSPP).

No AE or SAE were reported during the clinical trial. Based on results from IGAS performed at day 6, the safety of treatment was evaluated as “very good” or “good” by 93.9% (n = 62) and 6.1% (n = 4) of investigators in the DSPP + SoC group, and 77.8% (n = 49) and 22.2% (n = 14), of investigators in SoC alone group (Figure 6). The difference between the DSPP + SoC and SoC alone groups was statistically significant (*p* = 0.008). Confirming these excellent safety data, no additional concomitant treatment was required during the study.

## 4. Discussion

A key issue in children with tonsillitis-pharyngitis is the distinction between GABHS infections such as *Streptococcus pyogenes* and viral tonsillitis [6]. In fact, clinical symptom scoring systems (Modified Centor or McIsaac Scores) can help in identifying the disease, but, as several publications have pointed out, none of the available scoring systems is sufficiently accurate to identify GABHS pharyngitis with reasonable certainty [24]. Therefore, the diagnosis of GABHS infection should be confirmed by a laboratory test (either a rapid antigen detection test or culture). It is therefore extremely important that pediatricians can easily and quickly identify the infant population in which it is appropriate to perform the rapid test. The presence of more than two criteria in the Centor score (absence of cough, fever above 38°, enlarged lymph nodes, presence of tonsillar exudate, and age under 15 years) suggests the performance of the rapid test. In contrast, if the McIsaac score is greater than two (presence of rhinitis, cough, rhinorrhea, diarrhea, stomatitis, oral ulcers), it is more likely to be a viral infection [25]. In addition, it is not considered appropriate to administer the rapid test to children under the age of three, because the risk of acute articular rheumatism should be very low at this age. The present study included only children between 3 and 12 years of age who had suffered from ATR for no more than 48 h and had a negative rapid test for GABHS and SARS-CoV-2 infection. A negative culture of the nasal/pharyngeal exudate was required to rule out subjects affected by bacterial diseases.

The primary endpoint, TSS, showed a statistically significant difference between supplemented and unsupplemented patients at day 6. The difference was particularly evident in sore throat and erythema, both of which are highly subjective symptoms. Consequently, patient ratings of PGAE and IGAE measurements were most likely influenced by the marked improvement in these two symptoms.

The mechanisms of action of the components of DSPP and their role in viral diseases have been discussed in the medical literature. In particular, *Pelargonium sidoides* ex-tract was renowned for its immunomodulatory properties, mainly through gene expression modulation, resulting in increased cytokine expression. In the respiratory tract, these changes enhance ciliary beat frequency, mucus clearance, intracellular killing, and phagocytosis. In the respiratory tract, these changes enhance ciliary beat frequency, mucus clearance, intracellular killing, and phagocytosis [26]. Several studies [27] showed activity of honey against a broad spectrum of viruses, including adenovirus and influenza viruses. It was shown [28,29] that not only propolis has shown a marked anti-microbial activity, but also its single chemical compounds have pharmacologic effects with synergistic activity against viruses.

The anti-infectious properties of zinc are contingent upon its direct interaction with viral replication and the stimulation of antiviral immunity. Indeed, zinc interferes with a broad array of activating and inhibitory molecular pathways in the complex network that follows lymphocyte activation [30,31] (i.e., the regulation of nuclear factor of activated T-cells (NFAT) transcription factor expression).

As stated in the introduction, several studies have reported on the use of DSPP activity as an adjuvant treatment for viral diseases. The increasing interest of clinicians to find useful tools that could work adjunctly to avoid the need for antibiotic therapy, especially in children, is confirmed by the publication of a recent metanalysis on *Pelargonium sidoides* [32] and a systematic review on honey and propolis [33]. The analyzed 11 trials [32] showed that *Pelargonium sidoides* reduces the burden and speed up the cough relief. On the other hand, the Randomized Clinical Trials (RCTs) mentioned in [33] showed that honey and propolis as adjuvant treatments added to SoC improve respiratory symptoms and decrease viral clearance time in almost 500 patients affected by COVID-19.

Although several studies have focused their attention on the cellular and humoral immune mechanisms of action of the individual components of DSPP, it could be very interesting to perform a preclinical study in an animal model to investigate the DSPP compounds (both separately and as a whole) to identify their potential synergistic activities. Similarly, a future double-blind RCT versus placebo could fill the main limitation of this study, which, as can be easily imagined, was due to economic issues. We hope that the results of this study and the positive feedback from thousands of pediatricians could be an incentive to plan such a study.

Finally, considering that several DSPP components showed strong in vitro antibacterial activity [26,30,34], the tested product could also play a role as an adjuvant treatment to antibiotics in the pediatric population affected by bacterial tonsillitis. Of course, this hypothesis should be verified in future clinical trials. In our opinion, the positive results in terms of reduction in TSS total rating, significant improvement in symptoms (sore throat and pharyngeal erythema), and reduction in used doses of paracetamol clearly demonstrate that the administration of DSPP in conjunction with SoC produces a clinically beneficial effect in children affected by viral acute tonsillopharyngitis. Furthermore, the absolute safety of DSPP (no AEs during the entire duration of the trial) and optimal compliance are paramount when administering medications or food supplements to young children, as they fulfill the fundamental requirements for safe and effective use.

## Figures and Tables

**Table 1:** Demographic characteristics of the Per Protocol (PP) population by assignment group at the baseline visit. Standard of care (SoC); dietary supplement PediaFlù^®^ (DSPP).

		All	DSPP + SoC	SoC	*p*
Age (years)	N	129	66	63	
	Mean (SD)	5.52 (2.07)	5.8 (2.01)	5.22 (2.11)	ns
	Median	5	5.5	5	
	Range	3–10	3–10	3–10	
BMI (kg/m^2^)	N	129	66	63	
	Mean (SD)	16.41 (3.06)	16.71 (3.0)	16.1 (3.11)	ns
	Median	15.54	15.97	15.42	
	Range	10.42–30.26	10.42–25.69	11.76–30.26	
Height (cm)	N	129	66	63	
	Mean (SD)	114.51 (14.95)	116.56 (15.36)	112.37 (14.31)	ns
	Median	114	116	108	
	Range	89–160	89–160	90–148	
Weight (kg)	N	129	66	63	
	Mean (SD)	22.1 (8.29)	23.32 (8.74)	20.83 (7.66)	ns
	Median	20	22	19	
	Range	11–60	12–60	11–54	

ns: non-significant (*p* > 0.05) Mann–Whitney U tests.

**Table 2:** Number of doses of paracetamol used during the study. Standard of care (SoC); dietary supplement PediaFlù^®^ (DSPP).

	DSPP + SoC	SoC	*p*
N	66	63	
Mean (SD)	13.67 (4.34)	15.92 (3.02)	<0.01
Median	14	18	
Range	5–18	6–18	

## Data Availability

The study methods [22] (p. 4), has been deposited at protocols.io (source at: https://www.protocols.io/view/a-randomized-open-controlled-study-to-evaluate-the-cmtqu6mw.html, accessed on 6 December 2023), a platform for developing and sharing reproducible protocols. The description of the study protocol was recently published [23]. In addition, the full study protocol (final version 2.0, dated 3 March 2021) and the data sets analysed in the study are available from the corresponding author on reasonable request.

## Supplementary Materials

The following supporting information can be downloaded at: https://www.mdpi.com/article/10.3390/children12030345/s1, Supplementary File S1: Synopsis Pitch Deck; Supplementary File S2: The consent forms.

## Abbreviations

AE/SAE	Adverse Event/Serious Adverse Event
AOM	Acute Otitis Media
ATR	Acute Tonsillopharyngitis/Rhinopharyngitis
CRO	Contract Research Organization
DS	Dietary Supplement
DSPP	Dietary Supplement PediaFlù^®^
IGAE	Investigator Global Assessment of Efficacy
IGAS	Investigator Global Assessment of Safety
GABHS	Group A beta-hemolytic Streptococcus
ITT	Intention-To-Treat
NFAT	nuclear factor of activated T-cells
PGAE	Patient Global Assessment of Efficacy
PP	Per Protocol
SARS-CoV-2	Severe Acute Respiratory Syndrome Coronavirus 2
SD	Standard Deviation
RCT	Randomized Clinical Trials
SoC	Standard of Care
TSS	Tonsillitis Severity Score
URTI	Upper Respiratory Tract Infection
