# Supplementary material for: Open, Randomised, Controlled Study to Evaluate the Role of a Dietary Supplement Containing Pelargonium sidoides Extract, Honey, Propolis, and Zinc as Adjuvant Treatment in Children with Acute Tonsillopharyngitis
Source: Children (Basel). 2025 Mar 10;12(3):345. doi: 10.3390/children12030345 (PMC11941233; doi:10.3390/children12030345)
Supplement: Supplementary file 1 [file children-12-00345-s001.zip › children-3432862-supplementary File S1.pdf]

Open, randomised, controlled study to evaluate the role of a dietary supplement containing *Pelargonium sidoides* extract, honey, propolis, and zinc as adjuvant treatment in children with acute tonsillopharyngitis

Fabio Cardinale[a]*, Dionisio Franco Barattini[b], Alessandro Centi[c], Greta Giuntini[c], Maria Morariu Bordea[d], Dorina Herțeg[e], Luca Barattini[f], Cristian Radu Matei[g]

a: UOC di Pediatria e PS, Azienda Ospedaliero-Universitaria "Policlinico-Giovanni XXIII", Ospedale Pediatrico Giovanni XXIII - Università di Bari
b: Opera CRO, a TIGERMED company, Timișoara, Romania
c: Pediatrica Srl, Livorno, Italy
d: CMMF Dr. Morariu Bordea, Timișoara, Romania
e: CM Dr. Herțeg Dorina, Timișoara, Romania
f: TIGERMED Italy, Genova, Italy
g: CM Dr. Matei Crisian-Radu, Timișoara, Romania

Study Design

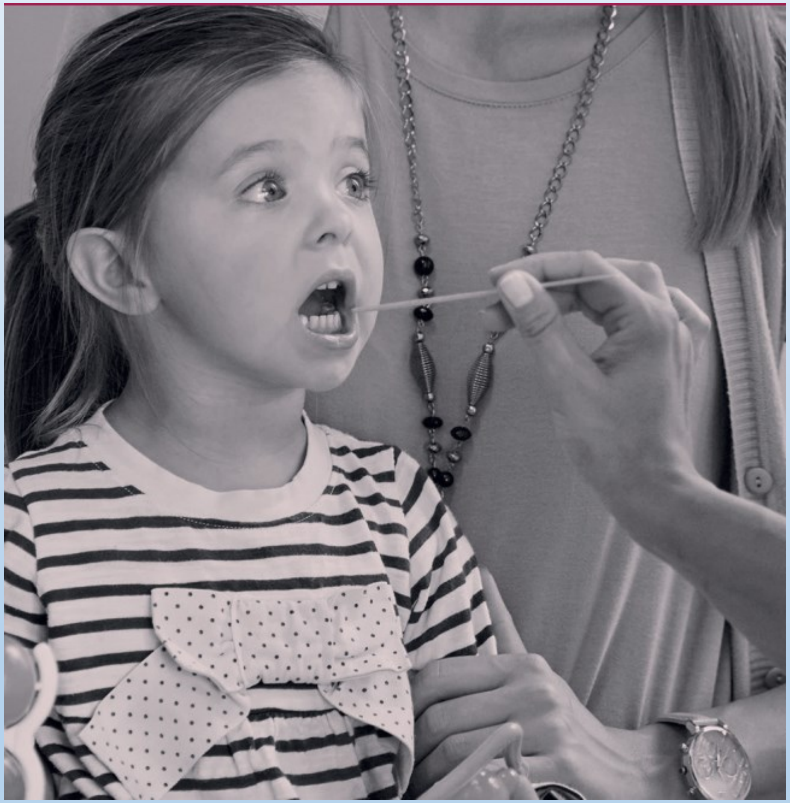

Randomized, open-label, controlled, multicenter trial
with a hypothesis of superiority

2 arms
129 children evaluable in total

3 Clinical Sites located in Romania

Introduction

Acute tonsillopharyngitis (ATP), often referred to as angina catarrhalis in Europe, affects mainly children, adolescent and young adults and represents one of the **most common reason** to consult a family physician.

ATP is highly prevalent, **seasonal** infective disorder characterized by an inflammation of the pharynx and the palatine tonsilis[1].

Children with non-streptococcal tonsillopharyngitis are often **over-treated with antibiotics**[2]**.**

1. *Shaikh N, Leonard E, Martin JM. Prevalence of streptococcal pharyngitis and streptococcal carriage in children: a meta-analysis. Pediatrics 2010 Sep;*
2. *Zanasi A, Lanata L, Saibene F, Fontana G, Dicpinigaitis PV, Venier V, De Blasio F. Prospective study of the efficacy of antibiotics versus antitussive drugs for the management of URTI-related acute cough in children. Multidiscip Respir Med 2016.*

Study population

135 screened children, **3-10 years**.
130 enrolled and randomized children, **3-10 years**
129 evaluable children, **3-10 years**

3 Pediatric clinics with extensive experience and wide catchment area in children ATR

Absence of pharyngeal exudate and/or Mc Isaac score 0-1 + negative rapid test for β-hemolytic streptococcus and SARS-CoV-2 identification.

The study is approved by the 3 local Ethical Committees (Timisoara, Romania)

Written informed consent was obtained by parents.

Study registered in Clinicaltrials.gov: NCT04899401

Inclusion criteria

- male and female (children 3 - 10 years old);
- acute tonsillopharyngitis/rhinopharyngitis (sore throat, catarrhal angina), duration of complaints ≤ 48 hours;
- negative rapid test for a β-haemolytic streptococcus or nasal and/or pharyngeal exudate culture and identification, and SARS-COV-2 infection;
- tonsillitis symptoms score (TSS) ≥ 8 points;
- written informed consent by both parents;
- willing to provide written informed consent (only for children ≥ 6 years.

Exclusion criteria

- evidence of lacunar or follicular angina;
- > two episodes of tonsillitis within the last 12 months;
- mandatory indication for therapy with antibiotics (e.g., abscess, septic tonsillitis);
- treatment with antibiotics within 4 months prior to study inclusion;
- increased haemorrhagic diathesis, chronic diseases (e.g., severe heart, kidne;
- close contact with SARS-COV-2 infected individuals in the last 10 days;
- known or suspected hypersensitivity to study medication;
- concomitant treatment potentially influencing study outcome;
- participation in another clinical study within the last 3 months.

Treatments

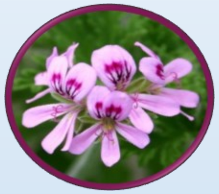

A 6-day study with administration of an extract of **Pelargonium sidoides** in pediatric patients with acute non-streptococcal tonsillopharyngitis showed a **clinically relevant decrease of disease symptomps** significantly superior to placebo[1].

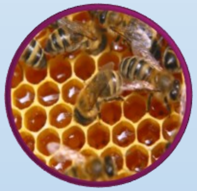

Zinc reduces the **average duration** of the **common cold** in healthy people assuming Zinc within 24 hours of onset symptoms[2].

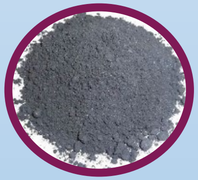

Propolis is administered as an add-on theapy in non-streptococcal pharyngitis[3].

1. *Bereznoy VV et al., Efficacy of extract of Pelargonium sidoides in children with acute non-group A beta-hemolytic streptococcus tonsillopharyngitis: a randomized, double-blind, placebo-controlled trial. Altern Ther Health Med 2003 Oct;*
2. *Singh M, Das RR. Zinc for the common cold. Cochrane Database Syst Rev 2011 Feb 16;*
3. *Di Pierro F, Zanvit A, Colombo M. Role of a proprietary propolis-based product on the wait-and-see approach in acute otitis media and in preventing evolution to tracheitis, bronchitis, or rhinosinusitis from nonstreptococcal pharyngitis. Int J Gen Med 2016.*

Treatments

Control group: Standard of Care (SoC).

vs

Interventional group: Dietary Supplement (DSPP) + SoC

- 5ml x 3/day orally for children < 6 years for 6 days
- 10ml x 3/day orally for children ≥ 6 years for 6 days

SoC
Product for nasopharyngeal liberation.
benzydamine hydrochloride
Paracetamol (or Ibuprofen)

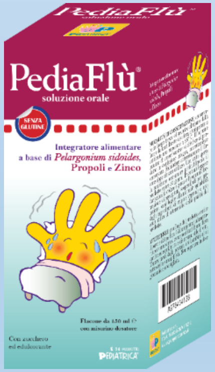

DSPP
Pelargonium sidoides,
Propolis (PropolNext® PLUS)
and ***Zinc***

Primary Outcomes

TSS (Tonsillitis Severity Score): change of TSS from baseline to final visit between groups

Number of treatment failures: rescue medicine (Ibuprofen or dosage of over 30 mg/kg/dose of Paracetamol) compared in the two groups

AE/SAE: incidence of Adverse Events/Serious Adverse Events

Secondary Outcomes

PGAE (Patient Global Assessment of Efficacy)

IGAE (Investigator Global Assessment of Efficacy)

IGAS (Investigator Global Assessment of Safety)

Investigational Product compliance

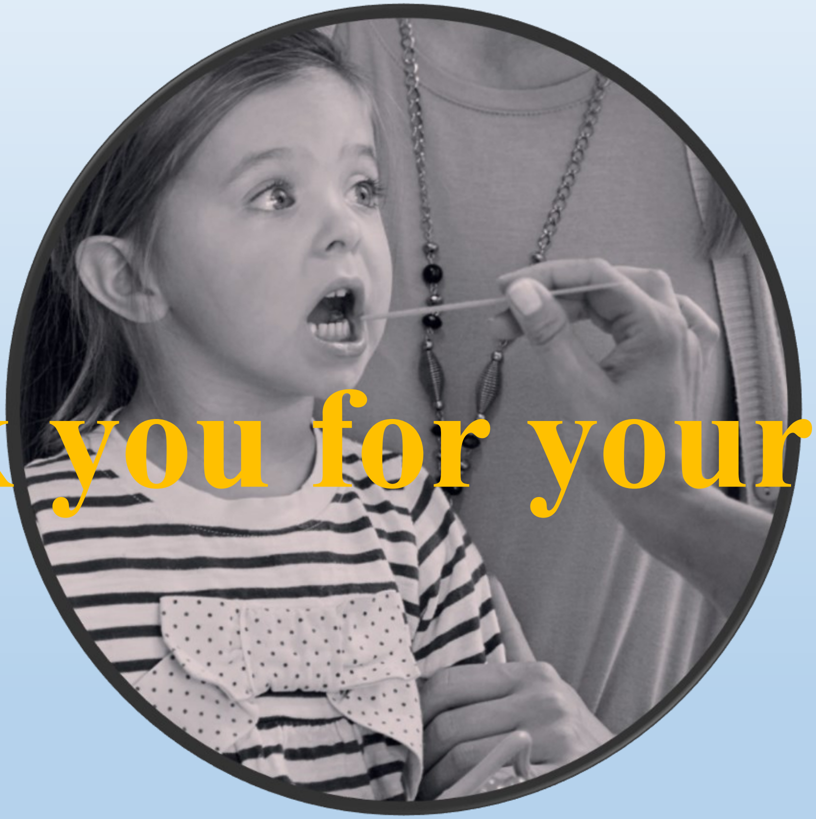

Thank you for your time
