# Supplementary material for: Open, Randomised, Controlled Study to Evaluate the Role of a Dietary Supplement Containing Pelargonium sidoides Extract, Honey, Propolis, and Zinc as Adjuvant Treatment in Children with Acute Tonsillopharyngitis
Source: Children (Basel). 2025 Mar 10;12(3):345. doi: 10.3390/children12030345 (PMC11941233; doi:10.3390/children12030345)
Supplement: Supplementary file 1 [file children-12-00345-s001.zip › children-3432862-supplementary File S2.pdf]

Study title: **A randomized open, two arms, controlled study to evaluate the efficacy and safety of PediaFlù® (dietary supplement) along with standard of care in children with acute tonsillitis / rhinopharyngitis versus standard of care only**

Protocol No.: **OPPED/0120/FS**
Version: **final 3.0 dated 16 Mar.2021**
Sponsor: **Pediatrica SRL**

Centre / _____
Principal Investigator Dr. _________________
Subject code ☐☐ ☐☐☐

PARENTS OR CAREGIVERS INFORMATION LEAFLET AND INFORMED CONSENT FORM

English Version

PARENTS OR CAREGIVERS INFORMATION LEAFLET

Dear parents and caregivers,

First of all, we would like to make sure that the language is clear to you and that you can read it effortlessly. Otherwise, please let your clinical investigation doctor know.

Your child is invited to take part in a clinical investigation addressing subjects that are suffer from acute tonsillitis / rhinopharyngitis, which will take place at _______________ and will be performed by Dr. ______________________ and his/her colleagues.

Your child has been chosen by us as a potential participant because he/she was diagnosed by the presence of acute tonsillitis / rhinopharyngitis.

Sore throat or acute tonsillopharyngitis, affects mainly children, adolescents and young adults and is one of the most common reasons to consult a family physician. While most patients complaining of sore throats have an infection, it has been estimated that fewer than 20% present with a clear indication for antibiotic therapy, i.e., are positive for haemolytic streptococcus. Children with non-streptococcal tonsillopharyngitis are often over-treated with antibiotics. The scientific literature currently published shows that the extract of Pelargonium sidoides has been shown to be effective in the treatment of diseases affecting the respiratory tract.

In this study, your child will receive PediaFlù® (dietary supplement) along with standard of care or standard of care only, for comparison purposes.

The purpose of this document is to explain what your child's participation in this clinical investigation entails so you and your child can decide whether she/he wish to take part or not. The next pages will describe the purpose of this investigation, what his/hers participation will imply, your and your child's rights and responsibilities. We kindly ask you to carefully read the following information and, if needed, to discuss them with trusted people (such as family, family doctor, or friends).

Your clinical investigation doctor will provide you with the following:

- One piece of this informed consent leaflet which contains answers to any potential questions you may have regarding this clinical investigation; should you have other questions your doctor will offer you answers about any aspect related to the investigation;
- One informed consent form.

We remind you that your child's participation is completely voluntary. Therefore, if you need further clarification or if you have more questions before taking part in this investigation, your clinical investigation doctor will address all your uncertainties and answer all your questions. If, after reading and understanding all information, you wish your child to take part in this investigation, you will be asked to sign the Informed Consent Form in duplicate. One copy will remain in your possession and the other will be kept by your doctor, while the medical records will be kept by the investigation centre: ________________________________.

Should you disagree to take part in this clinical investigation, there are other treatments available to your child. Your doctor can explain the available options to you in this case.

This clinical investigation will be conducted strictly abiding by the legislation regarding clinical investigations.

THE PURPOSE AND OBJECTIVES OF THIS CLINICAL INVESTIGATION

Who is sponsoring and conducting this research?

This research study is being sponsored by Pediatrica SRL (hereafter referred to as the study Sponsor) and is under the direction of your child's study doctor and the study staff for the investigational site. Pediatrica SRL is providing financial support to cover the cost of study-specific procedures performed during this study.

How many children will take part in the study?

Approximately 130 children and adolescents between 3 and 10 years of age with acute tonsillopharyngitis will take part in this study.

What are our obligations if my child takes part in this study?

If you decide to have your child take part in this study, you and your child will be required to do the following:

• Keep your child's study appointments and complete all study assessments.

If you and your child cannot attend an appointment, please contact study personnel (i.e., the study doctor or study staff) as soon as possible to schedule a new appointment.

• Inform study personnel about any symptoms, changes in medications, doctor's or nurse's appointments, or hospital admissions that your child may have had.

• Agree that your child will not participate in any other research study while in this study.

• Inform study personnel if you change your mind about your child participating in the study.

• Inform your child's other doctors that he or she is taking part in this study.

• Ask questions as you think of them.

If your child is allocated to receive study food supplement, you will be required to do the following:

• Ensure your child takes the study food supplement as instructed.

• Keep the study food supplement in a safe place, for your child’s use only, and away from direct access to it.

• Complete the administration diary as instructed.

• Return all study-related supplies, including any unused study investigational product.

STUDY CHARACTERISTICS

This study is defined as open and comparative.

Open study means that both you and your doctor during this clinical study will know if the investigational product will be administered during the study. Comparative study refers to the fact that one group has only standard of care and the other group has the investigational product, PediaFlù®, and standard of care administered to the subjects enrolled in this study and each subject will receive the appropriate dosage for his age. The efficacy of the investigational product will be assessed in comparison with the results obtained in the other group.

WHAT DOES YOUR STUDY PARTICIPATION ENTAIL?

Participant clinical centres and study duration

The subjects will be enrolled in centres referred as special care setting, located in Romania. The CRO will have previously evaluated the sites by means of feasibility questionnaires, pre-study visits and Investigators and staff CVs. In particular, the Investigators are GCP certified, have experience in clinical trials and a professional background of many years in treating subjects with acute tonsillitis / rhinopharyngitis.

The duration of the study will be 6 days from the beginning of the investigational product administration.

What will happen if my child takes part in this research study?

SCREENING ASSESSMENTS

Before your child begins the study, he or she will need to undergo the following tests or procedures to find out if he or she can be in the study. Some of these tests or procedures may be part of his or her regular medical care and may be done even if he or she does not take part in the study.

• Discussion of this study and review and signing of an Informed Consent Form. You may be asked to assist in reading the assent form to your child.

• Review of your child's medical history, and any treatments he or she is taking or has taken.

• Physical examination, including temperature, height and weight

INVESTIGATIONAL PRODUCT ADMINISTRATION

If your child meets all of the requirements for taking part in this study, he or she will be assigned to one of two groups: A or B. The assignment is random (like the toss of a coin), and your child could end up in either group. The chance of being randomly assigned to either Group A or Group B is 1:1. That is, for each subject randomly assigned to Group A (standard of care only), 1 patient will be assigned to Group B (PediaFlù® along with standard of care).

A description of each group is as follows:

• Group A: Subjects will be assigned to standard of care only. The study will be 6 days long.

• Group B: Subjects will be assigned to PediaFlù® along with standard of care. The study will be 6 days long.

PediaFlu® administration

PediaFlù® is given 3 times per day, by mouth. You will be given step-by-step directions and all the support you need on how to give the product to your child by the study doctor and study staff. PediaFlù® will need to be kept at an ambient temperature of up to 25°C, away from light and heat. Subjects who are assigned to PediaFlù® will need to keep an administration diary with the exact date and time that each dose of PediaFlù® was taken

and any altered or missed doses. The diary will be reviewed by the study doctor at each appointment.

The dose of PediaFlù® that your child receives will be based on your child's age:

- 5ml x3 times per day for children below 6 years and
- 10mlx3 times per day for children above 6 years

The study doctor will record any changes made to the original dose in the medication diary. Do not change the dosage by yourself, only the doctor can modify the dosage.

Standard of care

Standard of care for acute tonsillitis / rhinopharyngitis is focused on symptomatic treatment. It is recommended to your child by the study doctor, that can choose the version/ combination that is best for your child:

- Nasopharyngeal liberation of the through hydration with drinking fluids to support body fluid excretion, aspiration of secretions, NaCl solution for nasal irrigation, Nasal sprays with sea water, nasal spray with active compound (this is to be used only at special indication of the medical doctor)

- Throat spray (with or without anaesthetic) and/or throat drops (with or without anaesthetic)

- Acetaminophen (Paracetamol): at need, as antipyretic (>38,5C), painkiller 10mg/kg/dose, per need every 6-8 hours or according to the leaflet, maximum dosage 80 mg / kg / day;

In some cases Ibuprofen can be used as anti-inflammatory medicine. In the case of Ibuprofen usage all the administration will be documented. It will be used only if standard of care treatment not succeed in the amelioration of symptoms and with a dose of 10 mg/Kg/dose (dosage maximum 30-40 mg/Kg/day) and only after your doctor will confirm the possibility of administration.

You and your child will need to keep a medication diary and record any altered or missed doses. The diary will be reviewed by the study doctor at each appointment.

You will receive as standard of care the following treatment:

- Tantum Verde throat spray
- Panadol Baby
- Nurofen

Based on your child age and weight you doctor will give you a card with the administration guidelines.

ASSESSMENTS AND SAMPLES DURING THE STUDY

If the screening assessments show that your child can be in the study and if you and your child choose to take part, he or she will be enrolled in the study and will undergo the tests and procedures described below and in the following tables:

• At all appointments, your child may have a physical examination: temperature, height and weight.

• At all appointments disease assessment, concomitant treatments verification and Tonsillitis severity score (TSS) evaluation will be performed to continue to check on your child’s health.

• At all appointments, you and/or your child will need to tell the study doctor about how he or she has been feeling and report any symptoms or illnesses the child may have.

Schedule of observation points and assessments

Procedures	Visit 1	Visit 2	Visit 3	Visit 4
Days	-2 to-10	0	4	6
Informed consent	X			
Inclusion Criteria	X			
Exclusion Criteria	X	X	X	X
Rapid test for detection of beta-haemolytic streptococci or nasal and/or pharyngeal exudate culture	X			
COVID-19 rapid test	X			
Demographics and Medical history	X			
Physical examination	X	X	X	X
Disease assessment	X	X	X	X
Concomitant medication	X	X	X	X
TSS	X	X	X	X
Product delivery (food supplement)	X			
Product return (food supplement)				X
Subject diary delivery	X			
Subject diary return				X
Product accountability				X
IGAE				X
Adverse Events	X	X	X	X

How long will my child be in the study?

For subjects on both arms, the study length is approximately 6 days.

If your child has a serious side effect during the study, the study doctor will ask your child to visit the office for follow-up examinations, even after he or she has completed the regular study visits.

What are the possible side effects or risks of being in the study?

Your child may have side effects from the investigational products or procedures used in this study. Side effects can vary from mild to very serious and may vary from person to person. Everyone taking part in the study will be watched carefully for any side effects. However, Pediatrica SRL, the study doctor, and other doctors do not know all of the side effects that could occur. Your child’s study doctors may give your child medications to help lessen side effects. Many side effects go away soon after whatever is causing them is stopped. In some cases, side effects can be serious and may be long lasting or may never go away. You should talk to your child’s study doctor about any side effects your child has while taking part in the study.

SIDE EFFECTS KNOWN TO BE ASSOCIATED WITH PediaFlu

The following side effects are known to be associated with PediaFlu:

The potential risks for the involved subjects are those related to the oral P. sidoides extracts administration. Commonly cited side effects include stomach upset, nausea, heartburn, or worsening respiratory symptoms.

Pelargonium contains a substance known as coumarin that acts as an anticoagulant (blood thinner). Because of this, the subjects should avoid taking pelargonium with prescription anticoagulants like warfarin as this could lead to excessive bleeding. For the same reason, the administration of pelargonium should be stopped at least two weeks before a surgery or a dental procedure.

Pelargonium should also be used with caution in people with autoimmune diseases like psoriasis, rheumatoid arthritis, lupus, and autoimmune hepatitis. There is the risk to activate the antibodies that trigger autoimmune symptoms.

POSSIBLE RISKS AND DISCOMFORT ASSOCIATED WITH THE PRODUCT AND PROCEDURES IN THIS STUDY

Aside from the inconvenience of doctor visits, the study product and procedures in this study may involve the following foreseeable risks and discomforts:

• General: If you and your child agree to participate in the study and to whichever group your child is allocated, at any time that you have any concern about your child's health, you must seek medical advice without delay and contact the study doctor.

• Lack of effect of study product: It cannot be guaranteed that PediaFlu will definitely cure or even improve your child's acute tonsillitis. Your child's condition may remain unchanged or may worsen during his or her participation in the study.

• Unknown risks: It is possible that unknown complications and side effects could occur. You will be informed of any important new findings regarding PediaFlu or that may affect your willingness to continue your child's participation in the study. You will be asked to acknowledge that you have been informed of these findings in writing.

Are there benefits to taking part in the study?

There is no guarantee that your child will receive any benefits from this study, and taking part in this study may or may not cause your child's health to improve. Information from this study may help doctors learn more about PediaFlu,, and the treatment of children and adolescents with acute tonsillitis. This information may benefit other patients with acute tonsillitis condition in the future.

CONFIDENTIALITY OF DATA COLLECTED IN THE CLINICAL INVESTIGATION

All the ethical aspects related to this clinical investigation were examined and approved by the local Ethics Committee of the investigation centre before the start of this clinical investigation.

The processing of any personal information essential for your participation in this clinical investigation (indicated below) will be treated with the appropriate confidentiality and security procedures to ensure absolute confidentiality.

In accordance with the European Regulation No. 679/2016 for the protection of persons regarding data processing (called GDPR), your name and your child's name will not be disclosed outside the institution where the clinical investigation is conducted.

During your participation in this clinical investigation, the study doctor will replace your child's name with a special code that identifies her/him. Together with the clinical

investigation information, this code will be used by the Sponsor and its representatives for the purposes and in the terms described in this document.

All medical documents, any information and data required to be collected in accordance with the clinical investigation procedures shall be communicated and transmitted in material form, as appropriate, to the Sponsor.

Any collected data, except for the one above, shall not be revealed outside the clinical investigation centre. Any personal data resulting from the sponsor's project as well as their copies will be taught to the sponsor upon completion of the clinical trial.

Your and your child's personal data (name, surname, phone number, serial number and ID card number, personal numerical code, unique numerical code granted by the doctor in the clinical investigation, medical analysis results, previously anonymized by the clinical investigation doctor) will be processed by the Sponsor's representatives (clinical investigation monitors or staff of the research organization by contract, Opera CRO), in the sense that its representatives can view and verify their correspondence with the numerical code granted by the clinical investigation doctor.

By signing this consent, you agree that we keep the data collected from you in this clinical investigation for 15 years. You also have the right to withdraw this acceptance at any time.

All parties involved in conducting this clinical investigation (sponsor, sponsor's representatives and clinical investigation doctors involved who are subjected to professional secrecy) are aware of and comply with the provisions of the personal data protection legislation and assume all the consequences deriving from their capacity as empowered operator and data controllers, in consideration of those indicated in the table below:

**1.**	OPERATOR'S IDENTIFICATION	**SPONSOR: Pediatrica SRL**<br>Headquarter:
		**CENTRE: _______________**<br>**Doctor: _________________**<br>Headquarter: _______________________<br>Registering number: __________________<br>ID number: _________________
**2.**	IDENTIFICATION OF THE PERSON GUARANTEED BY THE OPERATOR	**OPERA CONTRACT RESEARCH ORGANIZATION SRL** (referred to as Opera)<br>Headquarter: Romania, Timiș county, Timișoara, 10 Cozia street<br>Registration number: J35/1760/2015<br>ID number: RO17446157
3.	INFORMATION REGARDING THE DATA PROTECTION RESPONSIBLE	**In the name of the Sponsor**: OPERA CRO<br><br>**In the name of Opera**: Ms. Mihaela Turlea<br>e-mail: dataprotection@operacro.com
4.	PROCESSING PURPOSE AND LEGAL BASIS	**Purpose**: According to the above, the Sponsor wants to carry out this clinical investigation, and in the case of positive clinical trial results, with benefits to subjects and society in general, to market the medical device on which this clinical investigation was conducted.<br><br>**The objective** of this clinical investigation is described above on page number 4<br><br>In this regard, a contract is concluded with the doctor, and the latter takes over and records the identification data of the

		participants in this clinical investigation, assigning them a 5-digit identification code.<br><br>In consideration of the commercial obligations assumed by Opera towards its client, the sponsor, Opera has access to personal data and verifies the correctness of the records carried out by the doctor, depending on the situations existing in the documents prepared by him / her. After verification, Opera prepares reports for the Sponsor in which indicates exclusively the numerical code of the participants in this clinical investigation.<br><br>**The legal basis of the processing**: art. 6 alin. 1 let. a from Regulation no. 679/2016 (GDPR)
5.	PROCESSED DATA	• Name and Surname: ____________________<br>• Phone number: ____________________<br>• Series and ID card number: ____________________<br>• Personal Numerical Code: ____________________<br>• Unique numerical code assigned by the doctor to the subject for anonymisation purposes<br>• The results of analysis, in an anonymized form by the doctor and having the numerical code assigned by the doctor, as correspondence
6.	PROCESSING TYPE	The personal data indicated in section 5 are collected by the clinical investigation doctor and viewed by Opera only for the purpose of verifying the correspondence accuracy with the assigned numeric code. Subsequently, Opera will prepare analysis reports for the Sponsor, in which it will only indicate the numerical code.

		All the activities carried out by Opera are provided under the signed contract with the sponsor (including the processing of personal data) under its authority and according to its indications. The only situation in which Opera has access to personal data is to verify the correctness of the records carried out by the doctor, depending on the situations existing in their documents. Thus, Opera moves to the investigation centre and verifies at the scene the performed records.<br><br>No personal data except the numerical code is collected, stored, transferred, modified or processed in any other way by Opera. Thus, Opera does not retrieve the documentation containing the subject, parents or cargivers personal data, does not make copies of it, does not write down or withhold information from documents that may represent personal data, except for the numerical code and the analysis results. Following the factual check, Opera records in its datasheets the 5-digit numerical code for each subject participating in this clinical investigation, which was established in the clinical investigation plan and applied by the doctor for data anonymization purpose, the analysis results and the final reports sent to the sponsor will contain only that code, and not the names or other identification data of the subjects to be able to link between that code and a certain subject. Therefore, neither in the Opera's or Sponsor's database will arrive subject's personal data who participated in this clinical investigation (targeted persons – with the exception of the numerical code and the analysis results) and so it is respected the principle of minimising data, being pseudonymised by the doctor itself.

7.	THE RECIPIENTS OF THE PROCESSING	Sponsor	Access to medical analysis results related to the unique numerical code assigned by the doctor to the targeted person for the purposes of anonymization and at the numeric code, as indicated in the reports prepared by Opera
		Doctor	Access to all the processed data indicated in section 5
		Opera	Access to all the processed data indicated in section 5, but the transmission and collection only of the numerical code and the medical results to the Sponsor, through the prepared reports
8.	PROCESSING DURATION AND STORAGE PERIOD	All data relating to this clinical investigation shall be kept at the clinical investigation centre for the period established according to ICH GCP (Good Clinical Practice guide), namely 15 years.<br><br>Opera can only keep a copy of the file containing the essential documents of the sponsor related to this clinical investigation (file called Trial Master file), according to the sponsor's request (not more than 15 years). All other documents, information and data shall be communicated and transmitted in material form, as appropriate, to the sponsor, the obligation to keep them not being in charge of Opera. Any personal data resulting from the sponsor's project as well as their copies will be taught to the sponsor upon completion of the clinical investigation. After this moment, Opera will delete all of this data, as well as all the copies made.<br><br>The sponsor will keep the reports received from Opera for 15 years.	

9.	TRANSFER TO THIRD COUNTRIES OR INTERNATIONAL ORGANIZATION	Opera will/will not make transfers of personal data to recipients located in third countries	
10.	YOUR RIGHTS AS A DATA SUBJECT	THE RIGHT TO ACCESS YOUR OWN DATA (ART. 15 GDPR)	You have the right to obtain from your doctor or sponsor, a confirmation if your child's personal data is processed, conveying a simple written request to the address indicated in section 3. If so, you have the right to access the information indicated in point 1–9.<br><br>The reply will be forwarded no later than 14 working days from the date of the request. Thus, you will be able to receive a free copy of the documents/part of documents that comprise strictly your personal data that is processed. Additional copies will be charged with 2 lei/page.
		THE RIGHT TO RECTIFY THE DATA (ART. 16 GDPR)	You have the right to correct the incorrect data processed by the operator, as well as to complete the incomplete ones.<br><br>Such steps shall be carried out without undue delay, no later than 14 working days from the date of the request in writing to the sponsor/doctor indicated in point 3.<br><br>If necessary, corrections and additions to the existing registers will be performed.
		THE RIGHT TO DELETE	You may be forgotten by requesting deletion of your and your child's data, upon expiry of the

		THE DATA (ART. 17 GDPR)	duration of the processing indicated in point 8, or at any time following the signature of the present, by submitting a written request to the Sponsor or doctor, to the addresses indicated in Point 3. The consequence of this will be to stop your child's participation in this clinical investigation with the deletion of data from records related to this clinical investigation, within a reasonable period of not more than 21 days.
		THE RIGHT TO RESTRICT DATA PROCESSING OR OPPOSE (ART. 18, 19 GDPR)	You have the right to obtain from the operator and from the recipient of the processing, the restriction of processing if:<br><br>▪ find that your and your child's data are not accurate (the restriction will operate during the verification of the situation);<br>▪ the processing is illegal and you object the deletion of data;<br>▪ there is no longer need for your personal data, but you ask them for a court;<br>▪ you have opposed the processing for the period during which it is verified that the interests of the operator prevail over those of you.<br><br>The operator and the recipient of the processing may continue to process the restricted data if the processing is necessary for the establishment, exercise or defence of a

			right in court or is necessary to protect/defend an individual or legal person, or it's justified by reasons of public interest, but only with your consent.
		RIGHT TO DATA PORTABILITY (ART. 20 GDPR)	You have the right to receive your and your child's processed data in a structured format that can be automatically and easily read. You can also request the transmission of that data to another operator.<br><br>The sponsor shall carry out these steps within 14 working days of the written request date submitted by you at the address indicated in point 3.
		THE RIGHT TO COMPLAIN TO THE COMPETENT AUTHORITY (ART. 13 ALIN. 2 LIT. D, ART. 77 -79 GDPR)	In the case of violation of the rights provided by the GDPR and indicated in section 10, you can submit an action to the court or a complaint at the competent supervisory authority of Romania. You may also formulate a remedy before the competent courts against the decision of that authority.
		THE RIGHT TO WITHDRAW THE CONSENT (ART. 7 ALIN. 3 GDPR)	If you no longer wish to consent to the processing of your and your child's data according to this, this will lead to the impossibility of continuing your child's involvement in this clinical investigation. No other consequence (whether financial or

			disciplinary) will be supported by you in this situation.<br><br>This right of withdrawal may be exercised at any time by submitting a written request to the sponsor/doctor indicated in point 3, and the steps necessary to delete<br>your and your child's personal data will be carried out at the express request according to the right to delete data (paragraph 10), within a reasonable period of not more than 21 days.<br><br>By signing this document, you express your consent to the processing of your and your child's personal data as indicated in section 5.
		THE RIGHT TO NOT BE SUBJECTED TO AN AUTOMATIC DECISIVE PROCESS (ART. 22 GDPR)	No automatic decisive processes apply to your and your child and your data.
		THE RIGHT TO OPPOSE (ART. 21 GDPR)	If you oppose to the processing of your and your child's data, the sponsor and the doctor will ensure compliance with this right.
		THE RIGHT TO BE INFORMED (ART.12, 13 GDPR)	If you refuse the processing of your and your child's data according to the present one, it will be impossible to involve your child's in this clinical investigation.

			If it is intended to further process your personal data for a purpose other than that of item 4, it will be provided information on the secondary purpose and all relevant additional information to you.<br><br>In the event of a restriction by European Union or an internal law, which affects those indicated herein, you will be properly informed.<br><br>Refusing to provide the above data makes it impossible for your child to participate in this clinical investigation.

Regarding the above, the undersigned

(name and surname) ____

declare that I understand all of the above and I have voluntarily and without constraint indicated my and my child's data as indicated in section 5 of the table.

I hereby declare that I expressly, freely, unequivocally and unconditionally give my consent for my child to participate in this clinical investigation and processing of my and my child's personal data according to the information mentioned above.

YOUR CHILD'S PARTICIPATION IS VOLUNTARY

It is important to know that the decision to participate in this investigation is entirely yours and your child's. Participation is free and completely voluntary. During the investigation

period you must not incur any costs for all medical or diagnostic procedures to which you will participate.

Please keep in mind that your participation is voluntary, you and/or your child will not be compensated for costs such as parking, bus, taxi, caretaker.

IF YOU DECIDE TO WITHDRAW FROM THE CLINICAL INVESTIGATION

You and/or your child may decide not to participate or to withdraw from the investigation at any time, even after the clinical examination has been carried out, without having to provide any explanation. Withdrawal does not disfavour or affect your relationship with the doctor or other member of the investigation team from __________________ centre and does not limit your right to receive the appropriate treatment.

In case of nonparticipation or withdrawal, your child shall receive all that is necessary for your child and the study doctor will continue to monitor.

If you and your child decide not to participate in the clinical investigation or withdraw from it later, the clinical data collected up to that point can be used for this clinical study, except for the case when you request in writing that the data cannot be used for the purpose of this study.

THE INVESTIGATION DOCTOR MAY DECIDE TO INTERRUPT THIS INVESTIGATION

The clinical investigation doctor can decide to stop the investigation for reasons related to your child's health. For example, if your child experience unusual reactions or effects, the doctor may decide to withdraw him/her from this investigation. In this case, you and your child will be immediately informed about other treatment options. Your child's health and safety are the main concern when the doctor takes a decision.

Furthermore, the sponsor of the investigation can cancel/suspend the clinical investigation at any moment for administrative reasons.

WHAT WILL HAPPEN IF DURING THE INVESTIGATION NEW INFORMATION ON THE ACUTE TONSILLITIS/ RHINOPHARYNGITIS APPEARS?

Should any new information appear (positive or negative), which affects the quantity or the frequency of risks/benefits of this clinical investigation or which can affect your and your child’s willingness to continue the investigation, you will be informed immediately. You will also receive a new Informed Consent Form where the new information will be presented, and you will need to sign a new Informed Consent Form to reconfirm your child’s participation in the clinical investigation.

THE ASSISTANCE YOUR CHILD WILL RECEIVE AT THE END OF THE INVESTIGATION

If your child needs additional treatment after his/ hers participation in this investigation, the doctor will offer him/her all the help and support needed.

YOUR AND YOUR CHILD’S RESPONSIBILITIES IN THE CLINICAL INVESTIGATION

You and your child must provide the doctor with all the information about your child’s medical history, medicines that your child has taken recently and currently. You and your child must also report whether your child is involved in other clinical investigations or if he/she participated in other studies during the previous months.

You and your child must carefully follow the instructions that will be provided by the doctor and show up for each of the scheduled visits.

It is yours and your child’s responsibility to refrain your child from taking any new medicine without permission from your study doctor. Disrespecting this requirement may damage your child’s health and, in certain cases, it may determine the interruption of the study.

It is yours and your child’s responsibility to inform the study physician of any supplement, medicine and/or new foods he/she will take during the study.

If this has taken place during the specified time frame, if it is the effect of the study period or if damage has been caused to your child's health it is your and your child's responsibility to immediately inform the study doctor, who will provide you with the necessary information and care.

YOUR CHILD'S RIGHTS IN THIS CLINICAL INVESTIGATION

If you agree that your child to take part in this clinical investigation, your child's rights will be protected by the provisions of the Helsinki Declaration (collection of ethical principles and reference point for the entire Medical Community):

- You and your child have the right to withdraw your/his/hers consent to this study at any point without explanations;
- You and your child have the right to stop taking part in this study at any point without penalties;
- Your child's participation in this study will not deny you of any of your child's rights to medical assistance.

Finally, should you or your child have any questions regarding your child's rights as study participant, you can contact your study doctor using the details below.

ANY QUESTIONS?

Should you need further clarification or information, please discuss with your study investigator. Take all the time you need to decide whether you wish your child to take part in this study or not.

Should you have any questions regarding your participation in this study or if you wish to speak to the investigator urgently during this clinical study (for example, during the night or for a sudden illness), **you can use the following phone numbers:**

Principal Investigator:

Doc. …………………………………….........

E-Mail ………………………………….......

Centre phone: …………………………….

Phone: ……………………………...........

and his/her colleagues:

Doc. ……………………………………....... Doc. …………………………….................

E-Mail ………………………………….........

Centre phone: ……………………………

Phone: ……………………………............

E-Mail …………………………………........

Centre phone: ……………………………..

Phone: ……………………………............

INFORMED CONSENT FORM

**Study title:**	**A randomized open, two arms, controlled study to evaluate the efficacy and safety of PediaFlù® (dietary supplement) along with standard of care in children with acute tonsillitis / rhinopharyngitis versus standard of care only**
**Protocol No.:**	**OPPED/0120/FS**
**Version:**	**final 3.0 dated 16 Mar.2021**
**Sponsor:**	**Pediatrica SRL**

Centre / ___ ______________________

Principal Investigator **Doc.** _________________

Subject No: ☐☐ ☐☐☐

DECLARATION OF THE PARTICIPANT'S PARENT OR CARGIVER IN THE CLINICAL INVESTIGATION

I have read and understood the information provided in the previous pages. I was given the opportunity to ask questions and I received satisfying answers. This is why I declare that I have been duly informed by Dr.. about the objectives, procedures, possible effects on my child's health, which my child's could benefit from by participating in **A randomized open, two arms, controlled study to evaluate the efficacy and safety of PediaFlù® (dietary supplement) along with standard of care in children with acute tonsillitis / rhinopharyngitis versus standard of care only,** protocol number OPPED/0120/FS.

In particular:

- I understand that my child's participation is voluntary;

- I understand that I and my child's are free to refuse to participate in this clinical investigation or to withdraw at any time, without indicating the reasons and without my child's medical or legal rights being altered;
- I am aware of the need to comply with the indications and procedures that have been discussed;
- I am aware of the benefits that may result from participation in the clinical investigation, as well as of the possible risks;
- I have been informed about how to obtain additional information and about who to contact in case of emergency;
- I have been informed that the sponsor may decide at any time to discontinue the clinical investigation;
- I agree that, during the clinical investigation period, an authorized person by the Sponsor will verify the correctness of the data provided by me from various sources;
- I agree that, during the clinical investigation period, a person authorized by the Sponsor will verify the correctness of the data provided by my child from various sources;
- I received a copy of this form.

We, as the parents of the child understand that we will be given a full copy of this form after it has been signed and dated. We have read it, or it has been read to us. We understand the information and have had our questions answered. We agree for our child to take part in this research study as described above and authorize my child's study doctor and his team to use and disclose (share) his or her health information as described in this Informed Consent Form.

Child (patient) name (print) ____

Parent/caregiver name (print) ____

Parent/caregiver name (print) ____

If applicable – Name of parent /caregiver's legally authorized representative (print)	Relationship to parent/caregiver
If applicable – Name of parent /caregiver's legally authorized representative (print)	Relationship to parent/caregiver
Parent/caregiver signature or signature of parent/caregiver's legally authorized representative	Date
Parent/caregiver signature or signature of parent/caregiver's legally authorized representative	Date

Your child's family doctor may be informed regarding your child's participation in the clinical study and the product that s/he is receiving. To do so, please mark one of the options:

☐ Yes, I agree ☐ No, I do not agree

IMPARTIAL WITNESS' DECLARATION

The undersigned (Name) .... I certify that Dr./Prof... explained to Mr. (Mrs)..

And Mr. (Mrs)...

the characteristics of this clinical investigation indicated in the attached file;

I also confess that

Mr. (Mrs.) ....

And Mr. (Mrs)...

had the chance to ask questions, if he/she felt it necessary, was informed and freely accepted to participate in this clinical investigation.

______________________________________ ______________

Impartial Witness' Signature, Date

THE INVESTIGATOR'S DECLARATION

I declare that I informed the subject about the purpose and the nature of the clinical investigation **A randomized open, two arms, controlled study to evaluate the efficacy and safety of PediaFlù® (dietary supplement) along with standard of care in children with acute tonsillitis / rhinopharyngitis versus standard of care only,** clinical investigation protocol version final 3.0 of 16 Mar.2021.

I also declare that I have answered to all the questions of the subject's parents/caregivers about the nature, commitment, procedures, benefits and risks of participating in this clinical investigation.

Enough elements were provided during the interview to assert that the subject's parents/caregivers understands the information provided and contained in this document and that they voluntarily agreed the child to participate in this clinical investigation.

____

Investigator's Name and Surname

____ _______________

Signature Date

Study title: **A randomized open, two arms, controlled study to evaluate the efficacy and safety of PediaFlù® (dietary supplement) along with standard of care in children with acute tonsillitis / rhinopharyngitis versus standard of care only**

Protocol No.: **OPPED/0120/FS**
Version: **final 3.0 dated 16 Mar.2021**
Sponsor: **Pediatrica SRL**

Centre / _____
Principal Investigator Dr. _________________
Subject code | | | | | |

OLDER CHILDREN INFORMATION LEAFLET

AND

OLDER CHILDREN ASSENT FORM

English Version

OLDER CHILDREN INFORMATION LEAFLET

Dear participant,

First of all, we would like to make sure that the language is clear to you and that you can read it effortlessly. Otherwise, please let your clinical investigation doctor know.

We are asking whether you would like to take part in a clinical investigation. Research is a way to test new ideas to see if we can do things better and help more people. Research helps us learn new things.

Before you decide if you want to take part in this study, it is important that you understand why the study is being done and what it will involve for you. So please consider this information carefully and talk about it with your family, doctor, or nurse if you want to.

If there is anything that you don't understand, then please ask the study doctor or the study staff.

Why is this study being carried out?

We are asking young people with acute tonsillitis / rhinopharyngitis to take part in this study. Acute tonsillitis / rhinopharyngitis is the name of the disease that is affecting your throat. The throat is a very important part of your body. Product called PediaFlù® is used as food supplement in children, to support the correct functionality of the respiratory parts of your body and the well-being of the body. It can therefore be used to help in common colds.

We want to find out whether this product also show benefit and are okay to use in children with sore throat.

PediaFlù® is a liquid, you should drink it 3 times a day, by mouth.

A group of people called a Research Ethics Committee have reviewed and approved this study to ensure that it is okay for children like you to take part in the study.

Subjects in this study will be divided into *two* groups. This is so we can compare children who are administrated PediaFlù® plus standard of care *and* those who are administrated only standard of care. We will be asking about *120* children between 3 and 10 years old from Romania to take part in the study. You will know what group you are in, but you cannot choose the group.

Do you have to take part?

Taking part in this study is your choice. You can say yes or no. If you decide to take part in the study, you can change your mind and leave the study whenever you want. Whatever you decide is okay. Your doctor will still take good care of you.

How long will the study last?

For both groups the study duration is 6 days. *Both* groups will return to the doctor for check-up visits. These visits will allow your study doctor to check on your health.

What will happen during the study?

If you decide you would like to take part in the study, the study doctor first needs to go through a checklist to ensure that this study is right for you. This process is called screening. During screening, the following procedures will be completed:

- You will be asked questions about how you are feeling, physically, any illnesses that you have had, and any medications that you may have taken or still take.

- It is important to know how your body reacts while you are in the research study. You will be examined, and various measurements will be taken, such as your weight, height, and temperature.

If, after all these tests and questions, the study doctor thinks this study is right for you, you will be put into one of *two* groups.

- Group A

- Group B

The group you end up in will be chosen by *equal* chance (like the *toss of a coin*). One child will end up in Group A and one in Group *B*.

If you are in Group A you will receive standard of care for 6 days,

If you are in Group *B*, receive standard of care plus PediaFlù® for 6 days.

After screening, during the product administration period, you will have to come to the doctor for three more appointments.

At all of your appointments, the study doctor will ask how you have been feeling and whether you have had to take any medications. You may be examined and various measurements will be taken, such as your weight, height, and temperature.

How do you take PediaFlù®?

PediaFlù® is given three times a day by mouth. A diary will need to be kept to record the exact date and time that each dose of PediaFlù® was taken. Your parent or caregiver will help you with this. The diary will be reviewed by the study doctor at each appointment.

Are there any risks or bad things that can happen from taking part in this study?

It is important that you tell the study doctor and your parent(s) if you experience any discomfort. You must also tell your study doctor if you take any new medication including medication available without prescription or "natural" medicines. Bellow are some of the possible things that could happen.

PediaFlù® contains a substance called P. sidoides extract.

The potential risks for the taking PediaFlù® are those relative to *P. sidoides* extracts. Some people taking PediaFlù® can feel unwell (side effects). Very common side effects have included stomach upset, nausea, heartburn, or worsening respiratory symptoms.

Pelargonium contains a substance known as coumarin that acts as an anticoagulant (blood thinner). Because of this, it should be avoided taking pelargonium with prescription anticoagulants like warfarin as this could lead to excessive bleeding. For the same reason,

the administration of pelargonium should be stopped at least two weeks before a surgery or a dental procedure.

Pelargonium should also be used with caution in people with autoimmune diseases like psoriasis, rheumatoid arthritis, lupus, and autoimmune hepatitis. Doing so may activate the antibodies that trigger autoimmune symptoms.

Are there any benefits to taking part in this study?

Your sore throat may improve from taking PediaFlù® with standard of care.

Taking part in the study may help future patients by providing important information about PediaFlù® and the treatment of children with acute tonsillitis / rhinopharyngitis.

Are alternative treatments available?

There may be other treatments available for your sore throat. Your doctor will go through these with you and your parent/caregiver so the right treatment choice can be made for you.

What will happen to all the information collected on you?

We will keep your information in confidence. This means we will tell only those who have a need or a right to know. Wherever possible, we will share only information that has your name and address removed. When you enroll in the study, your records will become part of the research database. Only the researchers, the Sponsor of the study, Sponsor representatives and collaborators, representatives of the Ethics Committee will have access to the data collected during this study. The data from all patients in the study will be examined as a whole and may be presented at scientific meetings or in medical journals. The Regulatory Authorities may inspect study records (which include individual medical records).

Information collected for this study will be kept as long as required by law, however no shorter than the period required by the Opera, which is 15 years from the end of trial.

What if you want to leave the study?

If you start the study and then later decide you don't want to take part any more, you need to tell the study doctor. You can leave the study at any time for any reason. You will still be looked after.

Who can answer your questions about the study?

You can ask questions at any time, but if you would like further information about the study, please contact the study doctor or a second member of his team at:

Dr ____

Tel. ____

or

Dr ____

Tel. ____

After discussing with your parents, you may choose to also notify your family doctor regarding your participation in this clinical trial. Please discuss this aspect with your parents and together with them you may choose whether to inform him/her or not.

As in any clinical research a health insurance (taken at …………….., an insurance company from …………..) was made for this study. Your parents have already been informed in detail about this aspect and you can ask them or your study doctor to further explain what this means.

Do you have to decide right now?

You do not need to make a decision straight away. Please think carefully about the information you have been given and talk it over with your family and your doctor.

OLDER CHILDREN ASSENT FORM

Study title: **A randomized open, two arms, controlled study to evaluate the efficacy and safety of PediaFlù® (dietary supplement) along with standard of care in children with acute tonsillitis / rhinopharyngitis versus standard of care only**

Protocol No.: **OPPED/0120/FS**

Version: **final 3.0 dated 16 Mar.2021**

Sponsor: **Pediatrica SRL**

Centre / _____

Principal Investigator **Dr. _________________**

Subject code ☐☐ ☐☐☐

DECLARATION OF THE PARTICIPANT IN THE CLINICAL INVESTIGATION

Child's Statement and Signature

Please read the statements below and circle "yes" or "no" for each.

I've read through the information on this form about this study.	Yes	No
Somebody has explained this study to me, and I understand what it is about.	Yes	No

I understand what will be involved for me in this study.	Yes	No
I've asked all the questions I want and know that I can ask questions at any time.	Yes	No
I've had my questions answered in a way that I understand.	Yes	No
I know that I can stop taking part in this study at any time.	Yes	No
I've had enough time to make my decision.	Yes	No
I am happy to take part in this study.	Yes	No

If the response to any of the statements above is "no" or you do not want to take part in the study, then you do not need to sign below.

If you are happy to take part in this study, then please sign your name below.

Child's signature	Print name	Date of signature

I, the undersigned, have fully explained the relevant information of Study OPPED/0120/FS to the subject named above. I will provide the subject and legal representative with a copy of this signed and dated assent form.

Investigator's signature	Print name	Date of signature

Study title: **A randomized open, two arms, controlled study to evaluate efficacy and safety of PediaFlù® (dietary supplement) along standard of care in children with acute tonsillitis / rhinopharyn versus standard of care only**

Protocol No.: **OPPED/0120/FS**
Version: **final 3.0 dated 16 Mar 2021**
Sponsor: **Pediatrica SRL**

Centre / _____
Principal Investigator **Dr. _________________**
Subject code

YOUNGER CHILDREN INFORMATION LEAFLET
AND
YOUNGER CHILDREN ASSENT FORM

English Version

(can be read to the child by parent, caregiver, or study staff.)

YOUNGER CHILDREN INFORMATION LEAFLET

Question: Do I have to take part?

Answer: No. It is up to you to decide. Your parents or caregiver will help you decide. No one will make you take part in this study. It is fine for you to say no. Nobody will be upset with you. We will still look after you.

Question: Can you tell me more about the product PediaFlù®?

Answer: PediaFlù® it is a liquid that you have to drink, 3 times a day for 6 days. The study doctor or nurse will teach your parent or caregiver how to give you the product.

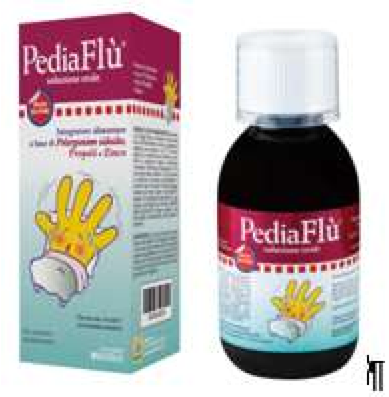

Children and teenagers in this study will be put into two different groups, one of the groups will be given standard of care and the other group standard of care together with PediaFlù®. That way, we can see if there is any difference between children who take PediaFlù® and children who don't.

Question : What will happen to me in the study?

Answer: You will need to come to the doctor for regular appointments. The study doctor or nurse will ask you how you are feeling, examine you, and take some measurements like your weight, height, and temperature.

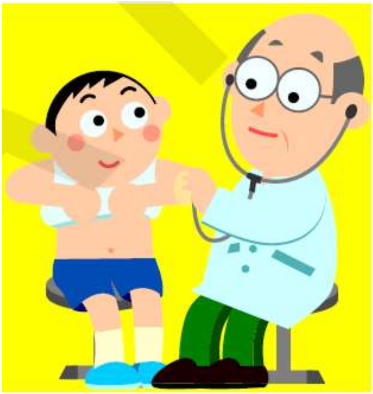

Question How long will I have to be in this study?

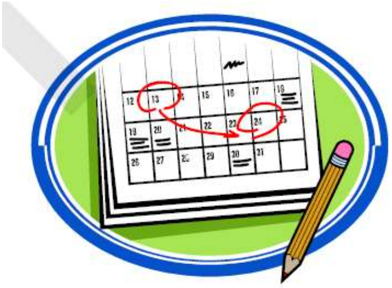

Answer: The study lasts for 6 days

If you change your mind and don’t want to be in the study any more, you just need to tell your parent or caregiver so that he or she can tell the study doctor. You can stop being in the study whenever you want; no one will be upset with you, and we will still look after you.

Question: What are the good things that could happen to me in this study?
Answer: We can’t promise that this study will help you, but the information we get might help us treat children with this product in the future.

Question: What are the bad things that could happen to me in this study?
Answer: Sometimes people don’t feel well when they have been given PediaFlù®. The study doctor or nurse will explain this to you and your parent or caregiver.

Question: What if I still have some questions? Do I have to decide now?
Answer: You can ask as many questions as you want, whenever you want. You don’t have to decide now if you want to be in the study. You can take your time and talk it over with your family and the study doctor or nurse.

Dr ____
Tel. ____

After discussing with your parents you may choose to also notify your family doctor regarding your participation in this study. Please discuss this aspect with your parents and together with them you may choose whether to inform him/her or not.

You might also want to know that this study was reviewed and approved by a team of men and women from the Local Ethics Committee whose job is to make sure that your rights are preserved and that you will be well taken care of.

In addition to this, a health insurance (taken at Ltd, an insurance company from) was made for this study. Your parents have already been informed in detail about this aspect and you can ask them or your study doctor to further explain what this means.

YOUNGER CHILDREN ASSENT FORM

Study title: **A randomized open, two arms, controlled study to evaluate the efficacy and safety of PediaFlù® (dietary supplement) along with standard of care in children with acute tonsillitis / rhinopharyngitis versus standard of care only**

Protocol No.: **OPPED/0120/FS**
Version: **final 3.0 dated 16Mar 2021**
Sponsor: **Pediatrica SRL**

Centre / _____
Principal Investigator Dr. _________________
Subject code ☐☐ ☐☐☐

DECLARATION OF THE PARTICIPANT IN THE CLINICAL INVESTIGATION

Child's Statement and Signature

Please read the statements below and circle "yes" or "no" for each.

I've got through the information on this form about this study.	Yes	No
Somebody has explained this study to me, and I understand what it is about.	Yes	No
I understand what will be involved for me in this study.	Yes	No

I've asked all the questions I want and know that I can ask questions at any time.	Yes	No
I've had my questions answered in a way that I understand.	Yes	No
I know that I can stop taking part in this study at any time.	Yes	No
I've had enough time to make my decision.	Yes	No
I am happy to take part in this study.	Yes	No

If the response to any of the statements above is "no" or you do not want to take part in the study, then you do not need to sign below.

If you are happy to take part in this study, then please sign your name below.

Child's signature	Print name	Date of signature

I, the undersigned, have fully explained the relevant information of Study OPPED/0120/FS to the subject named above. I will provide the subject and legal representative with a copy of this signed and dated assent form.

Investigator's signature	Print name	Date of signature
